# Associations of endogenous androgens and sex hormone-binding globulin with kidney function and chronic kidney disease

**DOI:** 10.3389/fendo.2022.1000650

**Published:** 2022-12-19

**Authors:** Lina Hui Ying Lau, Jana Nano, Cornelia Prehn, Alexander Cecil, Wolfgang Rathmann, Tanja Zeller, Andreas Lechner, Jerzy Adamski, Annette Peters, Barbara Thorand

**Affiliations:** ^1^ Institute of Epidemiology, Helmholtz Zentrum München - German Research Center for Environmental Health, Neuherberg, Germany; ^2^ Institute for Medical Information Processing, Biometry, and Epidemiology (IBE), Ludwig-Maximilians-Universität (LMU), Munich, Germany; ^3^ International Helmholtz Research School for Diabetes, Helmholtz Zentrum München, German Research Center for Environmental Health, Neuherberg, Germany; ^4^ Chair of Epidemiology, Institute for Medical Information Processing, Biometry and Epidemiology, Medical Faculty, Ludwig-Maximilians-Universität München, Munich, Germany; ^5^ Metabolomics and Proteomics Core, Helmholtz Zentrum München, German Research Center for Environmental Health, Neuherberg, Germany; ^6^ German Center for Diabetes Research (DZD), Partner Site Düsseldorf, Düsseldorf, Germany; ^7^ Institute for Biometrics and Epidemiology, German Diabetes Center (DDZ), Leibniz Center for Diabetes Research at Heinrich Heine Universität, Düsseldorf, Germany; ^8^ University Center of Cardiovascular Science, University Heart and Vascular Center Hamburg, Department of Cardiology, University Medical Center Hamburg, Hamburg, Germany; ^9^ German Center for Cardiovascular Research (DZHK), Partner Site, Hamburg/Kiel/Lübeck, Hamburg, Germany; ^10^ Medizinische Klinik und Poliklinik IV, Ludwig-Maximilians-Universität (LMU), München, Germany; ^11^ Institute of Experimental Genetics, Helmholtz Zentrum München, German Research Center for Environmental Health, Neuherberg, Germany; ^12^ Department of Biochemistry, Yong Loo Lin School of Medicine, National University of Singapore, Singapore, Singapore; ^13^ Institute of Biochemistry, Faculty of Medicine, University of Ljubljana, Ljubljana, Slovenia; ^14^ German Centre for Cardiovascular Research (DZHK), Partner Site Munich Heart Alliance, München, Germany; ^15^ German Center for Diabetes Research (DZD), Partner Site Munich-Neuherberg, Neuherberg, Germany

**Keywords:** testosterone (T), dihydrotestosterone (DHT), sex hormone-binding globulin (SHBG), chronic kidney disease, type 2 diabetes, kidney function

## Abstract

**Introduction:**

The role of endogenous androgens in kidney function and disease has not been extensively explored in men and women.

**Research design and methods:**

We analyzed data from the observational KORA F4 study and its follow-up examination KORA FF4 (median follow-up time 6.5 years) including 1293 men and 650 peri- and postmenopausal women, not using exogenous sex hormones. We examined the associations between endogenous androgens (testosterone [T], dihydrotestosterone [DHT], free T [fT], free DHT [fDHT], and T/DHT), with estimated glomerular filtration rate (eGFR) at baseline and follow-up, prevalent, and incident chronic kidney disease (CKD) adjusting for common CKD risk factors.

**Results:**

At baseline, 73 men (5.7%) and 54 women (8.4%) had prevalent CKD. Cross-sectionally, no significant associations between androgens and kidney function were observed among men. In women, elevated T (β=-1.305, [95% CI -2.290; -0.320]) and fT (β=-1.423, [95% CI -2.449; -0.397]) were associated with lower eGFR. Prospectively, 81 men (8.8%) and 60 women (15.2%) developed incident CKD. In women, a reverse J-shaped associations was observed between DHT and incident CKD (P_non-linear_=0.029), while higher fDHT was associated with lower incident CKD risk (odds ratio per 1 standard deviation=0.613, [95% CI 0.369; 0.971]. Among men, T/DHT (β=-0.819, [95% CI -1.413; -0.226]) and SHBG (P_non-linear_=0.011) were associated with eGFR at follow-up but not with incident CKD. Some associations appeared to be modified by type 2 diabetes (T2D).

**Conclusion:**

Suggestive associations are observed of androgens and SHBG with kidney impairment among men and women. However, larger well-phenotyped prospective studies are required to further elucidate the potential of androgens, SHBG, and T2D as modifiable risk factors for kidney function and CKD.

## 1 Introduction

Age-related kidney function decline can lead to CKD (1, 2). CKD development is accelerated by increasing prevalence of its risk factors such as obesity, smoking, hypertension and pre-eminently, T2D (2). As a well-known independent risk factor for cardiovascular and all-cause mortality, CKD represents a burgeoning silent epidemic straining healthcare systems (3). As CKD progresses faster in men (2), more focus has recently been placed on understanding the role of androgens in CKD development.

Aberrant androgen levels are a characteristic manifestation of CKD. Hypogonadism (a condition characterized by low T levels) is prevalent among men with CKD. Among women with CKD, androgen profiles are unclear (4), although women with metabolic syndrome show elevated T levels (5). Epidemiological reports in this context are inconsistent. Some observational cross-sectional (6) and prospective studies (7) report reduced kidney function with lower T levels in men, while others showed no differences (8, 9). In women, an observational prospective (9) and a Mendelian randomization (MR) study (10) found no relationships between T and kidney function. Further, despite being implicated in cardiovascular disease (CVD) (11) and sodium reabsorption (12), the role of DHT in kidney function has been barely evaluated (9, 13).

While bound to sex hormone-binding globulin (SHBG), a protein involved in sex hormone transportation, androgens are inactive. Meanwhile, free (unbound) androgens exert their effects in target tissues through androgen receptor binding (14). SHBG was prospectively-associated with better kidney function (9) and causally associated with lower CKD risk among men, but not among women (15). Few studies (9, 15) investigated the association between SHBG and kidney function in women. Therefore, additional investigations are needed.

Diabetic kidney disease (DKD) comprises 30-50% of CKD cases (16). T2D, representing an additional disease burden, could influence the relationship between androgens and kidney function. While sex-specific differences in androgen levels are evident in T2D (5), it remains unclear whether the putative associations between androgens and kidney function differ between person with and without T2D.

Therefore, the present study aimed to evaluate cross-sectional and prospective associations between levels of endogenous androgens and SHBG with measures of kidney function, as estimated by eGFR, in men and peri-/postmenopausal women from the general population. In addition, we aimed to assess whether any putative associations between androgens and kidney function were modified by the presence of T2D at baseline.

## 2 Methods

### 2.1 Study population

Data were obtained from the Cooperative Health Research in the Region of Augsburg (KORA) baseline (F4) (2006-2008) and follow-up (FF4) studies (2013-2014). Both studies are follow-up examinations of the KORA S4 study (1999-2001) conducted in Augsburg, Southern Germany, and two surrounding counties. The study design has been described previously in detail (17).

**Figure 1:**
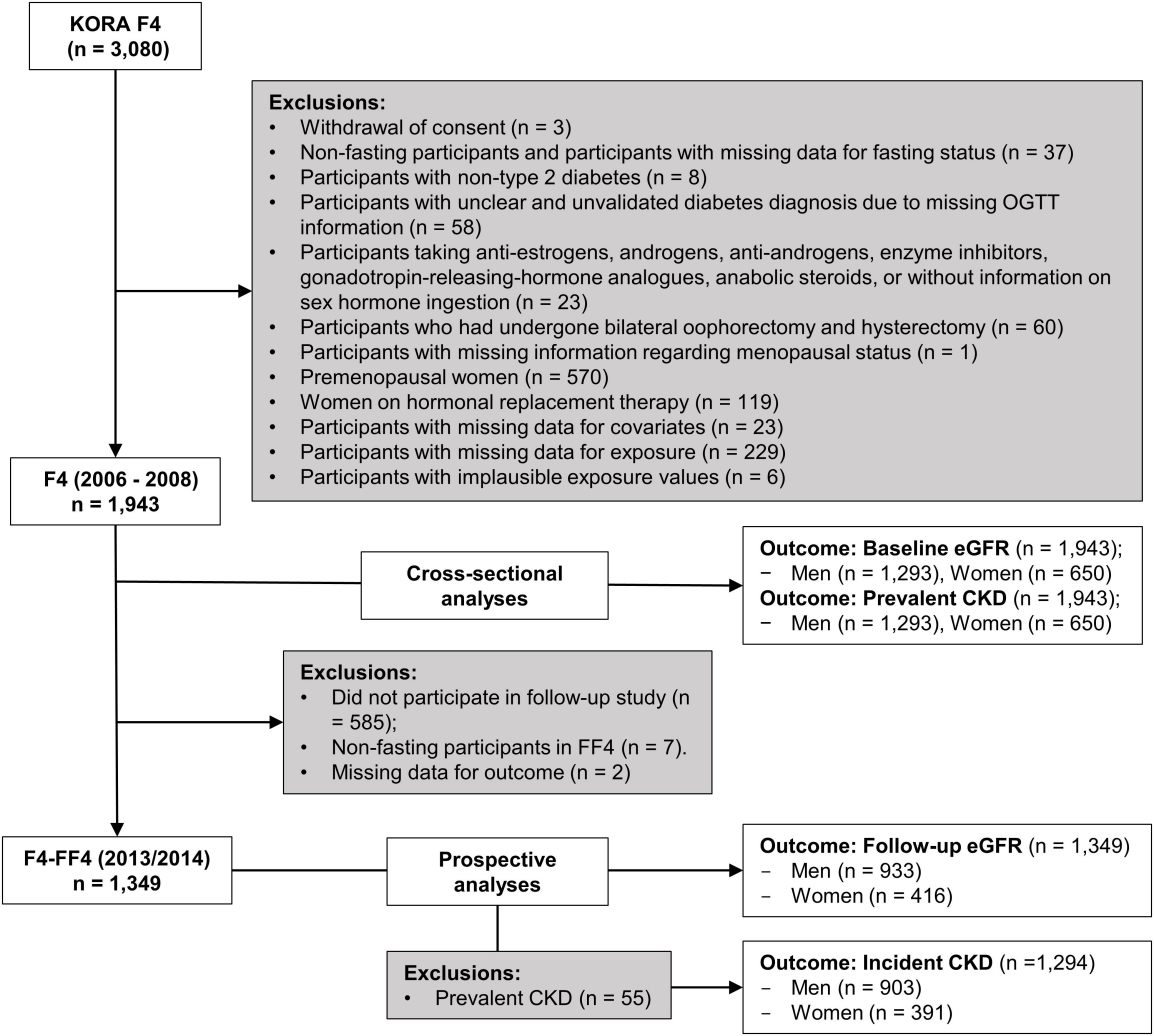
Exclusion flowchart.

The KORA F4 study included 3080 participants aged between 32 and 81 years, of which 2161 participated in KORA FF4. Three participants who withdrew consent and 570 premenopausal women were excluded from the analyses. After further exclusions as described in **Figure 1**, the final sample for the cross-sectional analyses comprised 1943 participants (1293 men and 650 peri-/postmenopausal women), while the prospective analyses sample comprised 1349 participants (933 men and 416 peri-/postmenopausal women) for follow-up eGFR and 1294 participants for incident CKD after exclusion of 55 participants with prevalent CKD.

### 2.2 Kidney function

Glomerular filtration rates were estimated based on serum creatinine concentrations using the Chronic Kidney Disease Epidemiology Collaboration formula (18). In the main analysis, prevalent CKD was defined as an eGFR <60 ml/min/1.73m² at baseline. Incident CKD was defined as eGFR <60 ml/min/1.73m² at follow-up in participants without prevalent CKD at baseline. Serum creatinine was measured in fresh serum with a modified Jaffe test (KREA Flex, Dade Behring) in F4 and the first part of FF4, whilst the standard Jaffe method was used in the second part of FF4 (Roche Cobas 8000 instrument). All measurements were calibrated to IDMS standards.

### 2.3 Androgen and SHBG quantification

Serum samples at baseline were collected and stored at -80°C until the analysis of androgens and SHBG. T and DHT were quantified in serum using liquid chromatography-electrospray ionization-tandem mass spectrometry and the AbsoluteIDQ Stero17 Kit (BIOCRATES Life Sciences, Austria) (19). The calibration, imputation, and normalization of sex hormone measurements are described in detail in the Supplementary Methods and Materials 1. For T measurements the intra-assay CV was 10.3%, the lower limit of quantification (LLOQ) was 0.35 nmol/L, and the upper limit of quantification (ULOQ) was 34.7 nmol/L. For DHT respective values were as follows: intra-assay CV: 11.1%, LLOQ: 0.04 nmol/L, ULOQ: 10.2 nmol/L. Intra-assay CVs were calculated using the means from five quality control samples and means over thirty-nine plates. T/DHT was calculated by dividing T concentrations by those of DHT. fT and fDHT were calculated based on measured T, DHT, SHBG, and serum albumin using the formula derived by Mazer (20). SHBG in serum was quantified using the ARCHITECT SHBG assay, a chemilumineschent microparticle immunoassay (Abbott Laboratories, USA). SHBG samples had an intra-assay CV of 4.3%. Serum albumin was quantified using immunophelometry (ALB Flex; Dade Behring, Germany).

### 2.4 Covariates

At baseline, non-high-density lipoprotein cholesterol (non-HDL-C) was calculated by subtracting HDL-C from total cholesterol to account for all LDL cholesterol types. Total cholesterol and HDL-C were measured in fresh serum by enzymatic methods (CHOL Flex and AHDL Flex, Dade Behring). C reactive protein (CRP) was quantified from frozen plasma using a high-sensitivity latex-enhanced nephelometric assay (BN II Analyzer, Dade Behring). Thyroid-stimulating hormone (TSH), free triiodothyronine (fT3), and free thyroxine (fT4) were quantified using immunochemiluminescent procedures (Dimension Vista System, Siemens, Germany).

Information on age, sex, waist circumference, prevalent cardiovascular diseases (CVD), anti-hypertensive medication usage, lipid-lowering medication usage, blood pressure, smoking status, alcohol consumption, and physical activity were assessed using a standardized interview, performed by trained medical staff (17). A participant was considered to have prevalent CVD if they had a history of either myocardial infarction, stroke, or angina pectoris. Participants’ medication use within seven days before the examination was assessed by trained medical staff using the IDOM database (21). Smoking status was categorized as never smokers, former smokers, and current smokers (≥1 cigarette a day). Physical activity was estimated through two separate four-category interview questions regarding the time spent per week on sport activities in summer and winter. Possible answers were (1) >2 hours, (2) 1-2 hours, (3) <1 hour, and (4) none. Participants who had a total score of <5, obtained by summing the numbers (1)-(4) relating to winter and summer, were considered to be ‘physically active’. Alcohol consumption was categorized into three groups: no consumption (0 g/day), moderate consumption (men 0.1-39.9 g/day, women 0.1-19.9 g/day), and high consumption (men ≥40 g/day, women ≥20 g/day) (22).

Previously known T2D was defined as a self-reported T2D diagnosis that was validated by a physician or medical chart review, or as self-reported current use of glucose-lowering mediation. Participants without known T2D obtained a standard 75g oral glucose tolerance test. Blood samples were taken without stasis after an overnight fast of ≥8 hours and 2 hours after glucose solution ingestion. Serum glucose was measured using hexokinase-G6PD (GLUFlex, Dade Behring, USA). Normoglycemia (NGT) (fasting glucose (FG) <6.1 mmol/L and 2 hour-glucose (2hG) <7.8 mmol/L), prediabetes [6.1 ≤ FG <7.0 mmol/L and 2hG <7.8 mmol/L (isolated impaired fasting glucose (IFG)] or FG <6.1 mmol/L and 7.8 ≤ 2hG <11.1 mmol/L (isolated impaired glucose tolerance (IGT)), or both (IFG and IGT)), and newly-diagnosed diabetes (FG ≥7.0 mmol/L or 2hG ≥11.1 mmol/L) were defined according to the 1999/2006 WHO criteria (23).

### 2.5 Statistical analyses

For baseline characteristics, categorical variables were presented as proportions (%), and continuous variables reported as mean (SD) or median (25^th^ and 75^th^ percentiles) for variables with normal and skewed distributions, respectively (**Table 1**). Natural log (ln)-transformations were applied to skewed variables to improve normality. Men and women were analyzed separately. Linear regression models were used to examine associations of baseline androgen and SHBG levels with continuous baseline and follow-up eGFR measures. Additionally, we examined the relationship between androgen and SHBG levels with prevalent and incident CKD using logistic regression. Exact time-to-event information regarding CKD manifestation during follow-up was unavailable, therefore logistic regression was used rather than survival analyses. Models with incident CKD as the outcome included men (n=903) and women (n=391) without prevalent CKD at baseline.

**Table 1:** Baseline characteristics.

	Men (n = 1293)	Women (n = 650)
**Age (years)^a^**	56 (13)	63 (9)
**BMI (kg/m²)^a^**	27.9 (4.2)	28.5 (5.3)
Waist circumference	98.5 (91.4, 106.1)	91.2 (82.5, 100.2)
**Systolic BP (mmHg)^a^**	128 (17.4)	120.8 (18.4)
**Diastolic BP (mmHg) ^a^**	77.7 (10.1)	73.5 (9.4)
**Antihypertensive medication (%)**	411 (31.8)	265 (40.8)
**Hypertension (%)**	469 (36.3)	278 (42.8)
**Prevalent cardiovascular diseases (%)**	123 (9.5)	70 (10.8)
**Total cholesterol (mmol/L)^a^**	5.52 (0.99)	5.97 (1.02)
**HDL-cholesterol (mmol/L)^a^**	1.30 (0.32)	1.58 (0.37)
**Non-HDL cholesterol (mmol/L)^a^**	4.21 (0.98)	4.40 (1.00)
**LDL-cholesterol (mmol/L)^a^**	3.56 (0.86)	3.75 (0.93)
**Triglycerides (nmol/L)^b^**	1.33 (0.93, 1.94)	1.21 (0.87, 1.63)
**C-reactive protein (mg/L)^b^**	1.09 (0.55, 2.39)	1.50 (0.75, 3.06)
**Lipid-lowering medication (%)**	187 (14.4)	102 (15.7)
Smoking status (%)		
Never	393 (30.4)	374 (57.5)
Former	639 (49.4)	195 (30.0)
Current	261 (20.2)	81 (12.5)
**Physically active (%)**	709 (54.8)	357 (54.9)
Alcohol consumption (%)		
None	256 (19.8)	263 (40.5)
Moderate ^c^	773 (59.8)	281 (43.2)
High ^c^	264 (20.4)	106 (16.3)
Baseline diabetes status (%)		
Normal glucose tolerance	896 (69.3)	426 (65.8)
Prediabetes	232 (18.0)	137 (21.1)
Type 2 diabetes	165 (12.7)	85 (13.0)
Kidney function		
Baseline eGFR (ml/min/1.73m²) ^a^	88.3 (16.2)	82.4 (15.7)
Follow-up eGFR (ml/min/1.73m²) ^a^	81.0 (16.4)	75.6 (16.1)
eGFR change (ml/min/1.73m²/year) ^b^	-1.18 (-2.22, -0.37)	-1.26 (-2.53, -0.41)
Baseline UACR (mg/g)	4.92 (3.14, 11.0)	7.23 (4.70, 12.9)
Follow-up UACR (mg/g)	4.12 (2.71, 8.30)	6.23 (4.08, 11.7)
Prevalent CKD ^d^ (%)	73 (5.7)	54 (8.4)
Incident CKD ^e^ (%)	81 (8.8)	60 (15.2)
Sex hormones		
Total T (nmol/L) ^b^	14.6 (11.4, 18.6)	0.62 (0.42, 0.88)
Free T (pmol/L) ^b^	191 (152, 228)	6.23 (4.20, 9.58)
DHT (nmol/L) ^b^	1.25 (0.90, 1.71)	0.18 (0.10, 0.29)
Free DHT (pmol/L) ^b^	6.85 (5.12, 8.81)	0.75 (0.41, 1.22)
T/DHT (unit) ^b^	11.9 (9.47, 14.4)	3.47 (2.18, 5.97)
SHBG (nmol/L) ^b^	48.2 (35.2, 65.4)	67.3 (48.3, 95.8)

^a^Measure of central tendency is presented as mean with corresponding standard deviation. ^b^Measure of central tendency is presented as median with corresponding 25th and 75th percentiles. ^c^Alcohol consumption defined as follows: moderate alcohol consumption (males 0.1-39.9 g/day and females 0.1-19.9 g/day), and high alcohol consumption (males ≥40 g/day and females ≥20 g/day). ^d^Defined as having an eGFR of <60ml/min/1.73m² at baseline. ^e^Defined as having an eGFR of <60 ml/min/1.73m² at follow-up. Those with prevalent CKD (n = 130; 73 males and 54 peri-/postmenopausal females) were excluded. BMI, Body mass index; CKD, Chronic kidney disease; DHT, Dihydrotestosterone; eGFR, Estimated glomerular rate; HDL, High density lipoprotein; LDL, Low density lipoprotein; SHBG, Sex hormone-binding globulin; T, Testosterone; UACR, Urinary albumin to creatinine ratio.

To enable sex-specific comparisons of association strengths across different sex hormones, effect estimates were calculated for a sex-specific one standard deviation (SD) increase in hormone concentrations. Models were adjusted for known risk factors for CKD: Age, waist circumference, systolic blood pressure, antihypertensive medication usage (yes/no), non-HDL-C, prevalent CVD (yes/no), lipid-lowering medication usage (yes/no), smoking status (never/former/current), physical activity (active/inactive), alcohol consumption (no/moderate/high), baseline diabetes status (NGT/prediabetes/T2D), and ln(CRP). Models with eGFR at follow-up and incident CKD as the outcome were additionally adjusted for baseline eGFR. Non-linearity was evaluated by including a non-linear term of hormone measurements in the models, and was visualized using restricted cubic splines. We evaluated the interaction between sex hormones and baseline diabetes status (i.e. NGT and prediabetes vs. T2D).

We performed additional sensitivity analyses: (1) We used a 3 SD cut-off for exclusion of participants with extreme androgen concentrations to ascertain the impact of extreme values on our estimates. (2) We further adjusted the models for TSH, fT3, and fT4 since thyroid hormones may impact androgen receptor expression and steroidogenesis (24, 25) and have been associated with kidney function (26–28). (3) We adjusted further with T in SHBG models to ascertain the independency of SHBG on assessed outcomes. (4) We excluded perimenopausal women as sex hormone levels can fluctuate during this phase.

Significance levels were based on two-sided tests, where p-values of <0.05 were considered to be statistically significant. Statistical analyses were performed using R (v4.0.5).

## 3 Results

Baseline characteristics of the study participants are presented in **Table 1**. At baseline, 127 (6.5%) participants [73 (5.7%) men, 54 (8.4%) women] had prevalent CKD. During a median follow-up time of 6.5 (25^th^, 75^th^ percentiles: 6.3, 6.6) years, 141 (7.2%) participants [81 (8.8%) men, 60 (15.2%) women] developed incident CKD. Due to the exclusion of premenopausal women in the present study, women were older than men; potentially explaining some baseline characteristic differences between men and women. Men had on average higher eGFR at baseline and follow-up, while women had steeper eGFR decline. As expected, total and free androgen levels as well as the ratio of T/DHT were considerably higher in men, while SHBG concentrations were higher in women.

**Figure 2:**
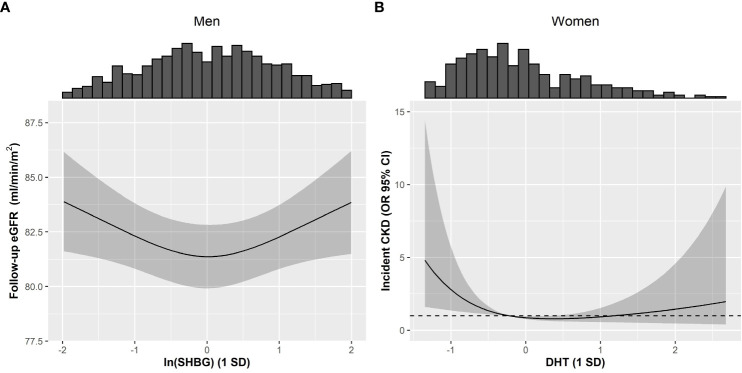
Restricted cubic splines describing non-linear associations between androgens or SHBG with kidney function in men and women of the KORA F4/FF4 study. Non-linear associations between **(A)** ln(SHBG) and follow-up eGFR in men, plus **(B)** between DHT and incident CKD in women. Non-linearity was investigated by introducing a quadratic term to the models for all exposures. Interaction between diabetes and DHT or SHBG was assessed using a 3-way interaction (i.e. DHT or SHBG*diabetes + (DHT or SHBG)^2^*diabetes). Non-linear associations were not modified by diabetes (P_interaction_: **(A)** 0.920 and **(B)** 0.948). Solid line represents the estimated spline function for follow-up eGFR and incident CKD, and the grey shaded area represents the respective 95% CI spline estimation. The histograms provide insight to the population density along ln(SHBG) and DHT values. Models were adjusted using Model 2, comprising of baseline eGFR, baseline age, waist circumference, systolic blood pressure, usage of antihypertensive medication, non-HDL cholesterol, prevalent cardiovascular disease, usage of lipid-lowering medication, smoking status, physical activity, alcohol consumption, diabetes status, and ln(CRP). CKD: Chronic kidney disease, CI: Confidence interval, CRP: C-reactive protein, DHT: Dihydrotestosterone, eGFR: Estimated glomerular filtration rate, HDL: High-density lipoprotein, OR: Odds ratio, SD: Standard deviation.

In fully-adjusted models, among men, we did not observe any significant associations between T, DHT, and SHBG with baseline eGFR and prevalent CKD. Excluding extreme values (>3 SD) in a sensitivity analysis did not discernibly change these observations (**Tables 2**, **S2**, **S4**). Prospectively, higher T/DHT was associated with lower follow-up eGFR (β_T/DHT_=-0.819, [-1.413; -0.226]). When extreme values were excluded, this association was attenuated to non-significance β_T/DHT_=-1.181 [-2.685; 0.322] (**Tables 3**, **S6**). Also, a U-shaped association between baseline SHBG and follow-up eGFR was observed (β_SHBG_=0.015, [-0.687; 0.717], β_SHBG_^2^ = 0.592, [0.133; 1.051]), P_non-linear_=0.011) (**Tables 3**, **S6**, **Figure 2A**). Further adjustment with T did not discernibly change the results (β_SHBG_=0.218, [-0.590; 1.026], β_SHBG_^2^ = 0.618, [0.156; 1.079]), P_non-linear_=0.009). Among 933 men, 921 had fT3, fT4, and TSH measurements. This association remained significant after further adjustment for thyroid hormones (β_SHBG_=0.051, [-0.655; 0.757], β_SHBG_^2 ^= 0.696, [0.102; 1.037]), P_non-linear_=0.017). Exclusion of extreme SHBG values (**Table S6**) did not discernibly alter this association. No significant associations between androgens, SHBG, and incident CKD were detected among men (**Table S8**).

**Table 2:** Cross-sectional associations of endogenous androgens and SHBG with baseline eGFR and prevalent CKD.

Exposure ^a^	Baseline eGFR β (95% CI)		Prevalent CKD OR (95% CI)	
	Men (n = 1,293)	Women (n = 650)	Men (n = 1,293)	Women (n = 650)
T	0.210 (-0.501 – 0.920)	**-1.305** **(-2. 290 – -0.320)** ^b^	0.810 (0. 597 – 1.081)	1.114 (0. 846 – 1.446)
fT	-0.034 (-0. 750 – 0.682)	**-1.423** **(-2. 449 – -0.397)** ^b^	0.765 (0.549 – 1.054)	1.166 (0. 874 – 1.539)
DHT	0.285 (-0. 395 – 0.965)	-0.197 (-1. 195 – 0.802)	0.964 (0. 663 – 1.327)	0.767 (0.484 – 1.132)
fDHT fDHT^2^	0.065 (-0. 611 – 0.740)	-0.358 (-1. 364 – 0.648)	0.910 (0. 571 – 1.353)	**0.549** **(0. 324 – 0.890)** **1.151** **(1.051 – 1.291)** ^b^
T/DHT	0.430 (-0. 227 – 1.086)	-0.405 (-1. 382 – 0.572)	0.790 (0. 486 – 1.098)	1.213 (0.976 – 1.473) ^b^
ln(SHBG)	0.372 (-0. 364 – 1.107)	-0.076 (-1. 190 – 1.037)	1.071 (0. 774 – 1.484)	1.027 (0.715 – 1.478)

a Non-linearity was investigated by introducing a quadratic term to the models for all exposures. Here, only significant (P<0.05) terms are reported. Quadratic terms, which were significant, were presented in this table along with corresponding linear terms (i.e. fDHT and fDHT^2^ with prevalent CKD in women).

b Number of participants after exclusion of androgen or SHBG measurements >3 SD above/below the mean; Men: T (n = 1,273), fT(n = 1270), DHT (n = 1,268), fDHT (n = 1,271), T/DHT (n = 1,279), and ln(SHBG) (n = 1,289). Women: T (n = 636), fT(n = 638), DHT (n = 637), fDHT (n = 642), T/DHT (n = 640), and ln(SHBG) (n = 648). Following estimates were revealed for baseline eGFR: β_T_ = -0. 770 (-2. 104 – 0.565), β_fT_ = -0.721 (-2. 117 – 0.675), OR_fDHT_ = 0. 571 (0.328 – 0.931), and for prevalent CKD: OR_T/DHT_ = 2.305 (1. 069 - 4.529).

β-estimates are per 1 sex-specific SD and adjusted using Model 2, comprising of baseline age, waist circumference, systolic blood pressure, usage of antihypertensive medication, non-HDL cholesterol, prevalent cardiovascular diseases, usage of lipid-lowering medication, smoking status, physical activity, alcohol consumption, diabetes status, ln(CRP). Interaction by diabetes status was assessed by entering the interaction as a multiplicative term (i.e. androgen or SHBG*diabetes) in Model 2. For models with a significant quadratic term (i.e. fDHT^2^), interaction was assessed using a 3-way interaction (i.e. fDHT*diabetes + fDHT^2^*diabetes). Prevalent CKD was defined as a baseline eGFR <60 ml/min/1.73m^2^. Bold values indicate statistical significance (p < 0.05).

CKD, Chronic kidney disease; CI, Confidence interval; CRP, C-reactive protein; DHT, Dihydrotestosterone; eGFR, Estimated glomerular filtration rate; HDL, High-density lipoprotein; SD, Standard deviation; SHBG, Sex hormone-binding globulin; T, Testosterone; T/DHT, T to DHT ratio.

**Table 3:** Prospective associations between endogenous androgens and SHBG with follow-up eGFR and incident CKD.

Exposure ^a^	Follow-up eGFR β (95% CI)		Incident CKD OR (95% CI)	
	Male (n = 933)	Female (n = 416)	Male (n = 903)	Female (n = 391)
T	-0.252 (-0.931 – 0.427)	-0.097 (-1.148 – 0.953)	0.851 (0.597 – 1.198)	0.932 (0.593 –1.432)
fT	-0.521 (-1.192 – 0.150)	-0.224 (-1.324 – 0.877)	0.875 (0. 594 – 1.272)	0.745 (0.429 – 1.231)
DHT DHT^2^	0.476 (-0.169 – 1.122)	0.310 (-0.755 – 1.376)	0.731 (0. 452 – 1.150)	**0.579** **(0. 350 – 0.938)** **1.230** **(0. 976 - 1.450)** ^c^
fDHT	0.156 (-0.486 – 0.797)	0.285 (-0.777 – 1.348)	0.828 (0. 475 – 1.394)	**0.613** **(0. 369 – 0.971)**
T/DHT	**-0.819** **(-1.413- -0.226)** ^b^	-0.215 (-1.190 – 0.759)	1.290 (0.662 – 2.519)	1.559 (0.692 – 3.605)
ln(SHBG) ln(SHBG)^2^	**0.015** (-0. 687 – 0.717) **0.592** **(0.133 - 1.051)**	0.558 (-0.620 – 1.736)	0.845 (0. 604 – 1.179)	1.207 (0.804 – 1.836)

a Non-linearity was investigated by introducing a quadratic term to the models for all exposures. Here, only significant quadratic terms (P<0.05) are reported. Quadratic terms which were significant were presented in this table along with corresponding linear terms (i.e. ln(SHBG) and ln(SHBG)^2^ with follow-up eGFR in men, as well as DHT and DHT^2^ with incident CKD in women). No other quadratic associations were observed in the main analyses.

b Number of participants after exclusion of sex hormone or SHBG measurements 3 SDs above/below the mean for follow-up eGFR; Men: T (n = 920), fT(n = 920), DHT (n = 913), fDHT (n = 915), T/DHT (n = 923), and ln(SHBG) (n = 931). Women: T (n = 408), fT(n = 410), DHT (n = 408), fDHT (n = 411), T/DHT (n = 410), and ln(SHBG) (n = 415). For incident CKD; Men: T (n = 890), fT(n = 891), DHT (n = 884), fDHT (n = 885), T/DHT (n = 893), and ln(SHBG) (n = 901). Women, T (n = 384), fT (n = 386), DHT (n = 383), fDHT (n = 386), T/DHT (n = 387), and ln(SHBG) (n = 390). After exclusion of measurements >3 SD, the association between T/DHT and follow-up eGFR in men was attenuated to non-significance (β = -1.181, [-2.685; 0.322]).

c Of 391 women, 375 had fT3, fT4, and TSH measurements. After adjusting for thyroid hormones, the non-linear association between DHT and incident CKD was attenuated to non-significance (β_DHT_=0.588, [0.343; 0.986], β_DHT_^2 =^ 1.188, [0.868; 1.429]), P_non-linear_=0.122)

ORs are per 1 sex-specific SD and adjusted using Model 2, comprising of baseline age, baseline eGFR, waist circumference, systolic blood pressure, usage of antihypertensive medication, non-HDL cholesterol, prevalent cardiovascular disease, usage of lipid-lowering medication, smoking status, physical activity, alcohol consumption, diabetes status, and ln(CRP). Interaction by diabetes status was assessed by entering the interaction as a multiplicative term (i.e. androgen/SHBG*diabetes) in Model 2. For models with a significant quadratic term (i.e. DHT^2^ and ln(SHBG)^2^), interaction was assessed using a 3-way interaction (i.e. DHT or ln(SHBG)*diabetes + DHT^2^ or ln(SHBG)^2^*diabetes). Incident CKD was defined as a follow-up eGFR <60 ml/min/1.73m^2^ in participants without prevalent CKD.

CKD, Chronic kidney disease; CI, Confidence interval; CRP, C-reactive protein; DHT, Dihydrotestosterone; eGFR, Estimated glomerular filtration rate; HDL, High-density lipoprotein; SD, Standard deviation; SHBG, Sex hormone-binding globulin; T, Testosterone; T/DHT, T to DHT ratio.

Among women, elevated T and fT were inversely associated with baseline eGFR (β_T_=-1.305, [-2.290; -0.320], β_fT_=-1.423, [-2.449; -0.397]). When extreme T and fT values were excluded, these associations did not persist (β_T_=-0.770, [-2.104; 0.565], β_fT_=-0.721, [-2.117; 0.675]) (**Tables 2**, **S3**). Additionally, a non-linear association was observed between fDHT and prevalent CKD (β_fDHT_=0.549, [0.324; 0.890], β_fDHT_^2 ^= 1.151, [1.051; 1.291]), P_non-linear_=0.007). Ultimately this non-linear association did not persist after excluding extreme fDHT values (P_non-linear_=0.963) (**Tables 2**, **S3**). Instead, we observed a significant linear association (OR_fDHT_=0.571, [0.328; 0.931]) (**Table S5**). A significant positive association between T/DHT and prevalent CKD (OR_T/DHT_= 2.305, 1.069; 4.529) was observed only after excluding extreme values (**Tables 2**, **S5**). Prospectively, no significant associations were observed between androgens, SHBG, and follow-up eGFR among women (**Tables 3**, **S7**). A reverse J-shaped association was seen between DHT and incident CKD (β_DHT_=0.579, [0.350; 0.938], β_DHT_^2 ^= 1.230, [0.976; 1.450]), P_non-linear_=0.025) (**Table 3**; **Figure 2B**). DHT values below the mean were inversely associated with incident CKD, while no association or possibly a very weak positive association was suggested for values above the mean (**Figure 2B**). This association remained significant after exclusion of extreme DHT values (β_DHT_=0.551, [0.334; 0.894], β_DHT_^2 ^= 1.689, [1.156; 2.491]), P_non-linear_=0.007) (**Table S9**). Among 391 women, 375 had fT3, fT4, and TSH measurements. The association between DHT and incident CKD did not persist after accounting for thyroid hormones (β_DHT_=0.588, [0.343; 0.986], β_DHT_^2^ = 1.188, [0.868; 1.429]), P_non-linear_=0.122). Additionally, an inverse association between fDHT and incident CKD was observed (OR_fDHT_=0.613, [0.369; 0.971]) – which did not appreciably change after excluding extreme fDHT values (**Tables 3**, **S9**). Among 386 women, 370 women had fT3, fT4, and TSH measurements. The association between fDHT and incident CKD remained significant after further adjustment for thyroid hormones (OR_fDHT_=0.613, [0.359; 0.993]). Notably, all observations among women did not significantly change when perimenopausal women were excluded.

Diabetes status was a significant effect modifier in several models. Among men, diabetes modified the association between T and follow-up eGFR (P_T×diabetes_=0.041). In men without diabetes, eGFR decreases (β_T(No diabetes)_=-0.425, [-1.130; 0.279]), whereas in men with diabetes, eGFR increases (β_T(Diabetes)_=1.709, [-0.679; 4.097]) as T levels increased. Among women, diabetes modified the association between T and baseline eGFR (P_T×diabetes_=0.014) and between DHT and prevalent CKD (P_DHT×diabetes_=0.001). Compared to women without diabetes, those with diabetes have steeper eGFR decline (β=-2.978, [-5.342; -0.614]) vs. β=-0.739, [-1.838; 0.361]) as T levels increased. However, due to the small sample size, we observed exceedingly wide CIs of the interaction term between DHT and diabetes status for prevalent CKD among women. Thus, stratified analysis was not performed.

## 4 Discussion

In the present study, we found several suggestive associations linking androgens and SHBG to kidney health among men and women. Among men, while no associations were observed between androgens, eGFR, and CKD, SHBG showed a U-shaped association with follow-up eGFR that was independent of T and not modified by diabetes status. In women, DHT showed a reverse J-shaped association with incident CKD, while elevated fDHT was significantly associated with lower CKD prevalence and incidence. Additionally, a higher T/DHT ratio was associated with higher CKD prevalence. Taken together, these findings suggest involvement of endogenous androgens and SHBG in CKD pathophysiology.

Our cross-sectional results in men regarding the lack of association of T and DHT with eGFR and CKD agree with the Diabetes Prevention Program Outcomes Study (DPPOS) that assessed the associations between endogenous androgens and kidney measures over 11 years in 2170 participants (889 men, 1281 women) (9). Similar observations were made in 1470 men from the Third National Health and Nutrition Examination Survey (8). The tendency towards an inverse association of T (and fT) with follow-up eGFR in the present study was concordant with a randomized controlled trial in 48 hypogonadal men, which showed that 3-month and 6-month T treatment lowered eGFR (29). In contrast to the above investigations, Kurita et al. (6) reported that elevated endogenous T was cross-sectionally associated with higher eGFR among Japanese men. Differing T levels among men attributed to genetic differences could explain this (30).

In the present study, women with extreme T and fT levels appear to have driven the inverse cross-sectional relationship with eGFR. The null association between T, fT, and baseline eGFR after excluding women with extreme T and fT levels is consistent to observations from Kim et al. (9). Hyperandrogenism is common among women with polycystic ovarian syndrome (PCOS) and T2D (5, 31). Higher BMI and waist circumference, as well as lower eGFR levels among women with extreme T and fT levels in the current study (data not shown) are consistent to previous reports of higher adiposity (32–34) and higher risk of PCOS-associated (35) and non-PCOS-associated kidney dysfunction (35–38). Androgen excess is associated with visceral fat accumulation (33, 39–41) and endothelial dysfunction (42–45), both of which can drive kidney dysfunction (46). This potentially explains our finding regarding the initial inverse association between T levels and eGFR; particularly for the extreme T and fT levels among women. Even though there is evidence that T may compromise kidney function in women (12, 47), the link between androgens and kidney function has not been extensively investigated. Thus, additional studies are required to better understand these associations among women.

We additionally observed during sensitivity analyses, that an elevated T/DHT ratio (higher T levels in regards to DHT) was associated with higher CKD prevalence among women, and that higher circulating levels of fDHT were associated with a lower prevalence and incidence of CKD. Considering that T and DHT levels, as well as T/DHT ratios are maintained at least partially by 5α-reductase (an enzyme responsible for converting T to DHT) (14), the possibility of sex-specific changes in the expression or activity of 5α-reductases during endocrine disorders merits further investigation.

The positive association between higher SHBG levels and eGFR at follow-up in men with SHBG levels above the mean, together with the tendency for an inverse association between SHBG and incident CKD was partially consistent to findings from an observational study (9) and an MR study that reported significant associations between genetically-predicted higher SHBG concentrations and lower CKD risk, independent of T (15). In women, a report (9) regarding SHBG was also consistent to our results as no association between SHBG levels and kidney function were observed. Notably, the abovementioned MR study (15) also did not find an association between genetically-predicted higher SHBG and CKD risk among women. Despite limited studies providing mechanistic insights linking SHBG and CKD, inflammation and insulin resistance could mediate this link. An *in vitro* experiment showed that SHBG suppresses inflammation - an effect not altered by simultaneous T or E2 supplementation (48). Based on further MR reports, genetically-predicted higher SHBG may lower insulin resistance and T2D risk in men and women (49–51). Moreover, in men, elevated fasting insulin has been linked to impaired kidney function, but not vice versa (52). Further studies that can provide sex-specific mechanistic insights to the SHBG-kidney relationship are warranted.

In the present study, we noted that adjustment for lipid levels and prevalent CVD consistently attenuated measures of association. Some plausible mechanisms linking androgens to kidney impairment include mediation through CV risk factors such as inflammation, hypertension (53), and hyperlipidemia (54). In patients with CKD, these risk factors are highly prevalent and contribute to atherosclerotic vascular disease, accounting for a majority of lesions that cause blood flow disruption to renal arteries (55). T can worsen atherosclerosis (56, 57), through its proinflammatory effects on the vascular system (58, 59). Hence, progression to kidney failure is accelerated. However, RCTs that attempted to assess CV safety associated with testosterone replacement therapy (TRT) in hypogonadal men (60–62) and postmenopausal women (63–65), showed no conclusive evidence that T supplementation is associated with increased CV risk (66). Albeit, these findings until now are derived from underpowered trials. Evidence concerning DHT is sparse. The few trials assessing the effects of DHT supplementation usually focused on DHT effects on prostate (67–69), rather than CV effects. Nevertheless, exogenous DHT has been shown to lower total and LDL-cholesterol, indicating favorable effects of DHT on traditional CVD risk factors in men (69).

RCTs to date have not investigated kidney outcomes following reinstatement of physiological androgen levels *via* TRT. Additionally, investigations in women are still lacking. Thus, further large-scale RCTs or population-based studies should assess the effects of androgens on kidney health in both sexes.

In the present study, baseline eGFR increases with higher T levels among men with T2D. Although T (and fT) have been inversely associated with T2D risk in epidemiological studies in men (70), glomerular hyperfiltration has been observed during early CKD stages among individuals with T2D (71); potentially explaining the opposing effects seen between eGFR and T among men with and without T2D. Moreover, diabetes-associated tubular hyperplasia and hypertrophy, as well as proximal tubular hyper-reabsorption reduces pressure in Bowman’s space. This perpetuates hyperfiltration (72). As shown in the current study population, women with T2D showed steeper eGFR decline compared to their counterparts without diabetes, and had higher T levels (5). As oxidative stress is more apparent in the hyperglycemic state (73), the cumulative effect of higher T levels and increased oxidative stress burden could accelerate the deterioration of kidney function. However, the link between sex hormones and DKD remains ambiguous and further investigations are needed.

To our knowledge, this is the first epidemiological study evaluating the role DHT in kidney function among women. Strengths of the current study include the prospective design, allowing the examination of prospective associations between endogenous androgens, SHBG, and changes in kidney function. As our study population was well-characterized, we were able to adjust for various relevant confounders. Further, mass spectrometry was used for androgen quantification. However, this study also has its limitations. Instead of a clinical CKD diagnosis, eGFR was used to define CKD. Sex hormone measurements were available only at baseline so we could not monitor the changes over time and evaluate their contribution to our outcomes of interest. However, single T measurements have been shown to be an adequate representation of the mean annual T levels (74). Next, we could not identify women with PCOS in our dataset as the information is unavailable. PCOS symptoms persist in postmenopausal women and could cause perturbations in sex hormone concentrations. Furthermore, we performed multiple comparisons as various associations between androgens, SHBG, and kidney function were examined. Due to the hypotheses-generating nature of our study, we did not adjust for multiple testing (75). Finally, despite adjusting for multiple risk factors for kidney disease, we cannot rule out any residual confounding or unmeasured confounders in the investigated associations.

## Conclusion

The results of the present study suggest that androgens and SHBG could be markers of kidney function impairment within a general population. Specifically, in men, we observed a U-shaped association between SHBG and follow-up eGFR, whereas in women, those with lower DHT levels may have an increased CKD risk. Conditions that are associated with abnormal hormonal profiles, such as T2D could alter the link between androgens and kidney function. Larger well-phenotyped prospective studies are required to further elucidate the potential of androgens, SHBG, and T2D as modifiable risk factors for kidney function.

## Data availability statement

The data analyzed in this study is subject to the following licenses/restrictions: Data are available upon reasonable request. Data collection in the KORA study is done in cooperation with the University Hospital of Augsburg. The data are subject to national data protection laws and restrictions were imposed by the ethics committee of the Bavarian Chamber of Physicians to ensure data privacy of the study participants. Therefore, data cannot be made freely available in a public repository. However, data can be requested through an individual project agreement with the Cooperative Health Research in the Region of Augsburg (KORA) *via* the online portal KORA.passt (https://helmholtz-muenchen.managed-otrs.com/external). Please contact the corresponding author, Barbara Thorand, in case of further questions. Requests to access these datasets should be directed to https://helmholtz-muenchen.managed-otrs.com/external.

## Ethics statement

The studies involving human participants were reviewed and approved by Bavarian Chamber of Physicians (Ethical Approval Number 06068). The patients/participants provided their written informed consent to participate in this study.

## Author contributions

LHYL and BT designed the study. CP, AC, TZ, WR, JA, AP, and BT contributed data. LHYL performed all data analyses with guidance from JN and BT, and is the guarantor of this work. Result interpretation was done by LHYL, JN, and BT. LHYL wrote the manuscript with guidance from JN and BT. All authors contributed to the article and approved the submitted version.
