# Supplementary material for: Associations of endogenous androgens and sex hormone-binding globulin with kidney function and chronic kidney disease
Source: Front Endocrinol (Lausanne). 2022 Dec 19;13:1000650. doi: 10.3389/fendo.2022.1000650 (PMC9807167; doi:10.3389/fendo.2022.1000650)
Supplement: Supplementary file 1 [file Table_1.pdf]

Supplementary Materials

Supplementary Materials and Methods 1 – Steroid Quantification of Hormones in Serum Samples

Steroid quantification in serum was performed at the Metabolomics Platform of the Genome Analysis Center, Helmholtz-Zentrum München. 19 steroids were quantified using an extended version of the Absolute*IDQ*™ Stero17 Kit and LC-ESI-MS/MS: aldosterone, androstenedione (androst-4-en-3,17-dione), androsterone, corticosterone, cortisol, cortisone, 11-deoxycorticosterone, 11-deoxycortisol, dehydroepi-androsterone (DHEA), dehydroepiandrosterone sulfate (DHEAS), dihydrotestosterone (DHT), estradiol (E2), estrone, etiocholanolone, 17α-hydroxyprogesterone, progesterone, testosterone (T), pregnenolone, pregnanediol (the last two steroids were assessed semi-quantitatively). Compound identification and quantification were based on scheduled multiple reaction monitoring measurements (sMRM). Sample preparation and LC-MS/MS measurements were performed as described by the manufacturer in manual UM-STERO17 [1]. Absolute*IDQ*™ Stero17 Kit methodology conforms to the "Guideline on Bioanalytical Method Validation" outlined by the EMEA [2], implying proof of reproducibility within a given error range. Analytical specification for LOD (limit of detection), LLOQ, and ULOQ (lower and upper limit of quantification), specificity, linearity, precision, accuracy, reproducibility, and stability were determined experimentally by Biocrates and are described in the manual AS-STERO17. A detailed method description has been published [3]. Method implementation in the laboratory is described as below:

Serum samples have been prepared in the group of the collaboration partner. All samples have been stored at -80°C until sample preparation for measurements. In the laboratory, 400 µl of ultrapure water were pipetted into each well of a 2 ml 96-well deep well plate. 20 µl of the internal standard mix was added to each well, except the blank cell. Thereafter, 250 µl of blank, calibration standards, quality control samples, and serum samples were pipetted into the distinct respective wells. The well contents were mixed by aspiration using robot-driven pipets. In between, the SPE (solid phase extraction) plate of the kit was conditioned successively with 1 ml of dichloromethane, followed by 1 ml acetonitrile, 1 ml methanol, and 1 ml ultrapure water. Except for sample loading, all SPE purification steps (conditioning, washing, drying, and eluting) were done by pressing solvents through the SPE plate using nitrogen and the positive pressure unit. The velocity was regulated by variation of the nitrogen pressure. After plate conditioning, the mixed samples were loaded onto the SPE plate. The samples dropped through very slowly by gravitation (1-2 drops per second). The SPE plate was washed with 500 µl water, dried for 1 h under nitrogen stream (58 psi). Steroids were subsequently eluted in two steps: 1) Two times with 500 µl dichloromethane into the same deep well plate (all steroids except DHEAS eluted), the eluate was dried each time for 20 min at 45 psi. 2) With 600 µl acetonitrile into another deep well plate. The first dichloromethane fraction was dissolved in 50 µl of methanol/water (25/75 v/v) and the plate was covered with a lid. To facilitate dissolving, the plate was treated for 1 min in an ultrasonic bath and afterward shaken for 5 min at 600 rpm. The second acetonitrile fraction was diluted with 400 µl of water and after covering the plate was treated like the dichloromethane fraction. Both plates were centrifuged at 50 x *g* and placed into the cooled autosampler

(10 °C) for LC-MS/MS measurements. The LC-separation of both fractions was performed using 470 ml ultrapure water and the content of three ampules of the kit as mobile phase A and acetonitrile/methanol/ultrapure water v/v/v 85/10/5 as mobile phase B. Steroids were separated on the HPLC column for Absolute*IDQ*™ Stero17 Kit combined with the precolumn SecurityGuard Cartridge C18 4 x 2 mm (for HPLC, Phenomenex Cat No. AJ0-4286).

HPLC grade solvents were used for sample preparation and measurements. Samples were handled using a Hamilton Microlab STARTM robot (Hamilton Bonaduz AG, Bonaduz, Switzerland) and a Waters Positive Pressure-96 Processor (Waters GmbH, Eschborn, Germany), besides standard laboratory equipment. Mass spectrometric analyses were using a QTRAP 5500 triple quadrupole system (Sciex Deutschland GmbH, Darmstadt, Germany) equipped with a 1260 Series HPLC (Agilent Technologies Deutschland GmbH, Böblingen, Germany) and a HTC PAL autosampler (CTC Analytics, Zwingen, Switzerland) controlled by the software Analyst 1.6.2. Data evaluation for quantification of metabolite concentrations and quality assessment was performed with the software MultiQuant 3.0.1 (Sciex) and the Met*IDQ*™ software package, which is an integral part of the Absolute*IDQ*™ Stero17 Kit. Metabolite concentrations were calculated using internal standards and reported in nM or ng/ml.

Supplementary Methods and Materials 2 – Data pre-processing of Testosterone and DHT measurements

The methods below were used for NA imputation and removal, normalizing the measurements to their plate effects, as well as for calibrating for batch effects for measurements of testosterone and DHT, where necessary.

i) NA removal and imputation

NA detection was conducted and removal was performed if more than 40% of steroids of all samples were labeled "NA" or "0" in the raw data set. For the raw data set, only estrone was detected to fulfill these criteria and was therefore subsequently removed from the data set. All other steroids were below 40% NA. These remaining NA or zero values were imputed by a minimum values replacement algorithm, implemented as follows: The minimum value - which could be measured for each metabolite from all plates combined - is gathered. This value is not allowed to be equal to zero as the export from the MetIDQ-System, in which the raw measurement data is translated to quantitative data, unfortunately, uses "NA" and "0" synonymously. To mitigate the effects which could be incurred by using these minimum values directly as imputation values, further steps are to be undertaken.

1. The minimum values are not used directly: they are divided by √2 to emulate their real concentrations being well below the minimally detected ones;

2. These newly calculated values, called "replacers", are then permuted randomly in a range of 0.75*replacer up to 1.25*replacer to mitigate the statistical effects of repeating the same number over and over again.

ii) Plate normalization

For each plate and metabolite, plate specific mean values ("plate means") are calculated. For this, the concentrations of each metabolite of the QC-2 samples (n = 5) are used. The plate means are then used to calculate an overall mean of all plates. These steps have to be performed for all metabolites (x_1, x_2, x_3, …, x_n) on all plates (n_1, n_2, n_3, …, n_j) of the data set.

$$Plate\ mean\ [metabolite\ (x)] = \frac{\sum C\ [metabolite(x)]}{N}$$

Where N = number of reference samples and C = concentration.

These plate means are then used to calculate the overall "means of all plates". This has to be calculated again for all metabolites (x_1, x_2, x_3, …, x_n) for each plate in the data set.

$$Overall\ mean\ [X] = \frac{\sum Means\ [X]}{N}$$

Where X = metabolite (x) of plates ($n \ldots n_j$) and N = number of plates ($n \ldots n_j$)

The plate factors are calculated for each plate separately, e.g. for plate 1 as follows:

$$Factor\ [Y] = \frac{Overall\ mean\ [X]}{Mean\ [Y]}$$

Where X = metabolite (X) of Plates and Y = Plate(n)[metabolite (X)]

In the final step of normalization, each metabolite concentration is multiplied with their corresponding plate factors, e.g. metabolite concentration of each sample on plate 1 is multiplied with plate factor 1.

$$Normalised\ [Y] = Factor\ [Y] \times Concentration\ [Y]$$

Where Y = Plate(n)[metabolite (X)]

In the last step, the duplicates from Batch 1 of the measurements are removed by averaging them.

ii) Calibration of batch effects of T and DHT

Regarding T and DHT measurements, serum samples from all KORA F4 participants were initially measured between January and November 2013 (batch 1). Due to measurement problems, they had to be repeated for 980 serum samples with the same methods described above between July 2017 and March 2018 (batch 2). To avoid batch effects, we used 175 duplicate measurements from the same participants of batches 1 and 2 to develop calibration formulas. The intercept and slope of Passing-Bablok regressions were used to calibrate batch 2 measurements with measures from batch 1.

Supplementary Methods and Materials 3 – Calculation of fT and fDHT

fT and fDHT were calculated using mass action equations as below [4]. Association constant values for respective sex hormones are described in Table 1.

$$Free\ X\left(\frac{pmol}{L}\right) = \frac{X_{total}\left(\frac{pmol}{L}\right)}{1 + \left(\frac{K\ SHBG_X\left(\frac{L}{mol}\right) \times SHBG\left(\frac{g}{L}\right)}{MW\ SHBG\left(\frac{g}{mol}\right)}\right) + \left(\frac{K\ ALB\left(\frac{L}{mol}\right) \times ALB\left(\frac{g}{L}\right)}{MW\ ALB\left(\frac{g}{mol}\right)}\right)}$$

Where X = T or DHT.

Table S1 - Variables and values (association constants and molecular weights) from Mazer 2019 used in the calculations:

**Variables**	**units**	**Remarks/values**
Testosterone (T)	nmol/L	NA
Dihydrotestosterone (DHT)	nmol/L	NA
Sex hormone-binding globulin (SHBG)	nmol/L	NA
Serum albumin	g/L	NA
**Association constants**		
SHBG to T (K $SHBG_T$) [4]	L/mol	1×10^9
Albumin to T (K ALB_T) [4]	L/mol	3.60×10^4
SHBG to DHT (K $SHBG_{DHT}$) [4]	L/mol	2.69×10^9
Albumin to DHT (K ALB_{DHT}) [4]	L/mol	6.36×10^4
**Molecular weights**		
Albumin (human)	g/mol or Da	66437
SHBG (human) [5]	g/mol or Da	90000

Table S2 – Cross-sectional associations between endogenous androgens with baseline eGFR in men of KORA F4.

Predictors	Outcome	All men (n = 1,293)		Exclusion of values more than 3 SDs above or below mean (n = hormone specific)[a]	
		β (95% CI)	p	β (95% CI)	p
**T**	Baseline eGFR	0.210 (-0.501 – 0.920)	0.563	0.102 (-0.723 – 0.927)	0.809
**fT**		-0.034 (-0.750 – 0.682)	0.925	-0.030 (-0.844 – 0.784)	0.942
**DHT**		0.285 (-0.395 – 0.965)	0.412	0.017 (-1.006 – 1.040)	0.974
**fDHT**		0.065 (-0.611 – 0.740)	0.851	-0.308 (-1.417 – 0.802)	0.586
**T/DHT**		0.430 (-0.227 – 1.086)	0.199	-1.165 (-2.708 – 0.379)	0.139
**ln(SHBG)**		0.372 (-0.364 – 1.107)	0.322	0.252 (-0.494 – 0.999)	0.508

[a] Number of participants after exclusion of sex hormone or SHBG measurements 3 SDs above/below the mean: T (n = 1,273), fT(n = 1270), DHT (n = 1,268), fDHT (n = 1,271), T/DHT (n = 1,279), and ln(SHBG) (n = 1,289).

β-estimates per 1 sex-specific SD calculated using linear regression models. Adjusted for baseline age, body mass index, systolic blood pressure, usage of antihypertensive medication, non-HDL cholesterol, prevalent cardiovascular disease, usage of lipid-lowering medication, smoking status, physical activity, alcohol consumption, baseline diabetes status, and ln(CRP).

Abbreviations: CI: Confidence interval, CRP: C-reactive protein, DHT: Dihydrotestosterone, eGFR: Estimated glomerular filtration rate, SHBG: Sex hormone-binding globulin, T: Testosterone.

Table S3 – Cross-sectional associations between endogenous androgens and SHBG with baseline eGFR in women of KORA F4.

Predictor	Outcome	All women (n = 650)		Postmenopausal women only (n = 558)		Exclusion of values more than 3 SDs above or below mean (n = hormone specific)[a]	
		β (95% CI)	p	β (95% CI)	p	β (95% CI)	p
**T**	Baseline eGFR	**-1.305 (-2.290 – -0.320)**	**0.009**	**-1.313 (-2.374 – -0.253)**	**0.015**	-0.770 (-2.104 – 0.565)	0.258
**fT**		**-1.423 (-2.449 – -0.397)**	**0.007**	**-1.396 (-2.497 – -0.296)**	**0.013**	-0.721 (-2.117 – 0.675)	0.311
**DHT**		-0.197 (-1.195 – 0.802)	0.699	-0.127 (-1.264 – 1.011)	0.827	-0.372 (-1.618 – 0.874)	0.558
**fDHT**		-0.358 (-1.364 – 0.648)	0.485	-0.307 (-1.453 – 0.839)	0.599	-0.330 (-1.559 – 0.899)	0.598
**T/DHT**		-0.405 (-1.382 – 0.572)	0.416	-0.439 (-1.450 – 0.573)	0.395	-2.041 (-5.262 – 1.180)	0.214
**ln(SHBG)**		-0.076 (-1.190 – 1.037)	0.723	-0.117 (-1.371 – 1.138)	0.855	-0.099 (-1.231 – 1.033)	0.864

[a] Number of participants after exclusion of sex hormone or SHBG measurements 3 SDs above/below the mean: T (n = 636), fT(n = 638), DHT (n = 637), fDHT (n = 642), T/DHT (n = 640), and ln(SHBG) (n = 648).

β-estimates are per 1 sex-specific SD and estimated using linear regression models. Adjusted for baseline age, waist circumference, systolic blood pressure, usage of antihypertensive medication, non-HDL cholesterol, prevalent cardiovascular disease, usage of lipid-lowering medication, smoking status, physical activity, alcohol consumption, baseline diabetes status, and ln(CRP).

Abbreviations: CI: Confidence interval, CRP: C-reactive protein, DHT: Dihydrotestosterone, eGFR: Estimated glomerular filtration rate, SHBG: Sex hormone-binding globulin, T: Testosterone.

Table S4 – Cross-sectional associations between endogenous androgens and SHBG with prevalent CKD in men of KORA F4

Predictors	Outcome	All men (n = 1,293)		Exclusion of values more than 3 SDs above/below mean (n = hormone specific)[a]	
		OR (95% CI)	p	OR (95% CI)	p
**T**	Prevalent CKD	0.810 (0.597 – 1.081)	0.162	0.903 (0.645 – 1.254)	0.545
**fT**		0.765 (0.549 – 1.054)	0.105	0.809 (0.568 – 1.150)	0.240
**DHT**		0.964 (0.663 – 1.327)	0.834	0.895 (0.578 – 1.359)	0.611
**fDHT**		0.910 (0.571 – 1.353)	0.671	0.978 (0.592 – 1.578)	0.929
**T/DHT**		0.790 (0.486 – 1.098)	0.262	1.011 (0.556 – 1.711)	0.970
**ln(SHBG)**		1.071 (0.774 – 1.484)	0.679	1.081 (0.780 – 1.504)	0.640

[a] Number of participants after exclusion of sex hormone or SHBG measurements 3 SDs above/below the mean: T (n =1,273), fT(n = 1,278), DHT (n = 1,268), fDHT (n = 1,271), T/DHT (n = 1,279), and ln(SHBG) (n = 1,289).

Prevalent CKD defined using eGFR <60 ml/min/1.73m^2. ORs are per 1 sex-specific SD and estimated using logistic regression models. Adjusted for baseline age, waist circumference, systolic blood pressure, usage of antihypertensive medication, non-HDL cholesterol, prevalent cardiovascular disease, usage of lipid-lowering medication, smoking status, physical activity, alcohol consumption, baseline diabetes status, and ln(CRP).

Abbreviations: CI: Confidence interval, CRP: C-reactive protein, DHT: Dihydrotestosterone, eGFR: Estimated glomerular filtration rate, SHBG: Sex hormone-binding globulin, T: Testosterone.

Table S5 – Cross-sectional associations between endogenous androgens and SHBG with prevalent CKD in women of KORA F4.

Predictor	Outcome	All women (n = 650)		Postmenopausal women only (n = 558)		Outliers >3 SDs above/below mean excluded (n = hormone specific)[b]	
		OR (95% CI)	p	OR (95% CI)	p	OR (95% CI)	p
**T**	Prevalent CKD[a]	1.114 (0.846 – 1.446)	0.425	1.111 (0.845 – 1.439)	0.434	1.160 (0.756 – 1.740)	0.483
**fT**		1.166 (0.874 – 1.539)	0.287	1.167 (0.876 – 1.536)	0.282	0.886 (0.541 – 1.398)	0.616
**DHT**		0.767 (0.484 – 1.132)	0.222	0.770 (0.486 – 1.136)	0.227	0.651 (0.384 – 1.051)	0.094
**fDHT**		0.895 (0.579 – 1.281)	0.585	0.893 (0.578 – 1.280)	0.579	**0.571 (0.328 – 0.931)**	**0.035**
**T/DHT**		1.213 (0.976 – 1.473)	0.056	1.205 (0.970 – 1.462)	0.064	**2.305 (1.069 – 4.529)**	**0.019**
**ln(SHBG)**		1.027 (0.715 – 1.478)	0.883	1.032 (0.719 – 1.484)	0.863	1.024 (0.712 – 1.474)	0.899

[a] Prevalent CKD defined using baseline eGFR <60 ml/min/1.73m^2.

[b] Number of participants after exclusion of sex hormone or SHBG measurements 3 SDs above/below the mean: T (n = 636), fT(n = 638), DHT (n = 637), fDHT (n = 642), T/DHT (n = 640), and ln(SHBG) (n = 648).

ORs are per 1 sex-specific SD and estimated using logistic regression models. Adjusted for baseline age, waist circumference, systolic blood pressure, usage of antihypertensive medication, non-HDL cholesterol, prevalent cardiovascular disease, usage of lipid-lowering medication, smoking status, physical activity, alcohol consumption, baseline diabetes status, and ln(CRP).

Abbreviations: CI: Confidence interval, CKD: Chronic kidney disease, CRP: C-reactive protein, DHT: Dihydrotestosterone, eGFR: Estimated glomerular filtration rate, OR: Odds ratio, SHBG: Sex hormone-binding globulin, T: Testosterone.

Table S6 – Prospective associations between endogenous androgens with follow-up eGFR in men of KORA F4.

Predictors	Outcome	All men (n = 933)		Outliers >3 SDs above/below mean excluded (n = hormone specific) [a]	
		β (95% CI)	p	β (95% CI)	p
**T**	Follow-up eGFR	-0.252 (-0.931 – 0.427)	0.466	-0.525 (-1.303 – 0.252)	0.185
**fT**		-0.521 (-1.192 – 0.150)	0.128	-0.727 (-1.507 – 0.053)	0.068
**DHT**		0.476 (-0.169 – 1.122)	0.148	0.504 (-0.524 – 1.531)	0.336
**fDHT**		0.156 (-0.486 – 0.797)	0.634	0.451 (-0.676 – 1.577)	0.433
**T/DHT**		**-0.819 (-1.413 – -0.226)**	**0.007**	-1.181 (-2.685 – 0.322)	0.123
**ln(SHBG) + ln(SHBG)^2**		0.015 (-0.687 – 0.717)	0.967	-0.003 (-0.709 – 0.702)	0.993
		**0.592 (0.133 – 1.051)** [b]	**0.011**	**0.631 (0.151 – 1.112)** [b]	**0.010**

[a] Number of participants after exclusion of sex hormone or SHBG measurements 3 SDs above/below the mean: T (n = 920), fT(n = 920), DHT (n = 913), fDHT (n = 915), T/DHT (n = 923), and ln(SHBG) (n = 931).

[b] β-estimates presented for linear and quadratic terms of ln(SHBG) due to significant quadratic term. Non-linearity was investigated by introducing a quadratic term to the models for all exposures. Here, only significant quadratic terms ($P<0.05$) are reported.

β-estimates are associated with 1 sex-specific SD increase in sex hormones. Models were adjusted baseline age, baseline eGFR, waist circumference, systolic blood pressure, usage of antihypertensive medication, non-HDL cholesterol, prevalent cardiovascular disease, usage of lipid-lowering medication, smoking status, physical activity, alcohol consumption, baseline diabetes status, and ln(CRP).

Abbreviations: CI: Confidence interval, CKD: Chronic kidney disease, CRP: C-reactive protein, DHT: Dihydrotestosterone, eGFR: Estimated glomerular filtration rate, SHBG: Sex hormone-binding globulin, T: Testosterone.

Table S7 – Prospective associations between endogenous androgens and SHBG with follow-up eGFR in women of KORA F4/FF4.

Predictor	Outcome	All women (n = 416)		Postmenopausal women only (n = 342)		Outliers >3 SDs above/below mean excluded (n = hormone specific)[a]	
		β (95% CI)	p	β (95% CI)	p	β (95% CI)	p
**T**	Follow-up eGFR	-0.097 (-1.148 – 0.953)	0.855	-0.111 (-1.211 – 0.989)	0.843	0.443 (-0.925 – 1.811)	0.525
**fT**		-0.224 (-1.324 – 0.877)	0.690	-0.216 (-1.384 – 0.953)	0.717	0.192 (-1.301 – 1.685)	0.801
**DHT**		0.310 (-0.755 – 1.376)	0.567	0.431 (-0.824 – 1.685)	0.500	0.321 (-0.983 – 1.624)	0.629
**fDHT**		0.285 (-0.777 – 1.348)	0.598	0.551 (-0.705 – 1.806)	0.389	0.407 (-0.869 – 1.683)	0.531
**T/DHT**		-0.215 (-1.190 – 0.759)	0.665	-0.112 (-1.101 – 0.877)	0.823	-0.316 (-3.540 – 2.907)	0.847
**ln(SHBG)**		0.558 (-0.620 – 1.736)	0.352	0.352 (-0.968 – 1.672)	0.600	0.682 (-0.506 – 1.869)	0.260

[a] Number of participants after exclusion of sex hormone or SHBG measurements 3 SDs above/below the mean: T (n = 408), fT(n = 410), DHT (n = 408), fDHT (n = 411), T/DHT (n = 410), and ln(SHBG) (n = 415).

β-estimates are per 1 sex-specific SD and calculated using linear regression models. Adjusted for baseline age, baseline eGFR, waist circumference, systolic blood pressure, usage of antihypertensive medication, non-HDL cholesterol, prevalent cardiovascular disease, usage of lipid-lowering medication, smoking status, physical activity, alcohol consumption, baseline diabetes status, and ln(CRP).

Abbreviations: CI: Confidence interval, CKD: Chronic kidney disease, CRP: C-reactive protein, DHT: Dihydrotestosterone, eGFR: Estimated glomerular filtration rate, T: Testosterone, SHBG: Sex hormone-binding globulin.

Table S8 – Prospective associations between endogenous androgens with incident CKD in men of KORA F4/FF4.

Predictors	Outcome	All men (n = 903)		Outliers >3 SDs above/below mean excluded (n = hormone specific)[a]	
		OR (95% CI)	p	OR (95% CI)	p
T	Incident CKD	0.851 (0.597 – 1.198)	0.361	0.851 (0.597 – 1.198)	0.361
fT		0.875 (0.594 – 1.272)	0.492	0.875 (0.594 – 1.272)	0.492
DHT		0.731 (0.452 – 1.150)	0.187	0.731 (0.452 – 1.150)	0.187
fDHT		0.828 (0.475 – 1.394)	0.490	0.828 (0.475 – 1.394)	0.490
T/DHT		1.290 (0.662 – 2.519)	0.448	1.290 (0.662 – 2.519)	0.448
ln(SHBG)		0.845 (0.604 – 1.179)	0.322	0.845 (0.604 – 1.179)	0.322

[a] Number of participants after exclusion of sex hormone or SHBG measurements 3 SDs above/below the mean: T (n = 890), fT(n = 891), DHT (n = 884), fDHT (n = 885), T/DHT (n = 893), and ln(SHBG) (n = 901).

Incident CKD defined using follow-up eGFR <60 ml/min/1.73m^2 in participants without prevalent CKD. ORs are per 1 sex-specific SD and estimated using linear logistic regression. Adjusted for baseline age, baseline eGFR, waist circumference, systolic blood pressure, usage of antihypertensive medication, non-HDL cholesterol, prevalent cardiovascular disease, usage of lipid-lowering medication, smoking status, physical activity, alcohol consumption, baseline diabetes status, and ln(CRP).

Abbreviations: CI: Confidence interval, CKD: Chronic kidney disease, CRP: C-reactive protein, DHT: Dihydrotestosterone, eGFR: Creatinine-based estimated glomerular filtration rate, OR: Odds ratio, T: Testosterone, SHBG: Sex hormone-binding globulin.

Table S9 – Prospective associations between endogenous androgens and SHBG with incident CKD in women of KORA F4/FF4.

Predictor	Outcome	All women (n = 391)		Postmenopausal women only (n = 317)		Outliers >3 SDs above/below mean excluded (n = hormone specific)[a]	
		OR (95% CI)	p	OR (95% CI)	p	OR (95% CI)	p
**T**	Incident CKD	0.932 (0.593 – 1.432)	0.753	0.951 (0.609 – 1.454)	0.822	0.932 (0.593 – 1.432)	0.753
**fT**		0.745 (0.429 – 1.231)	0.271	0.757 (0.437 – 1.252)	0.299	0.745 (0.429 – 1.231)	0.271
**DHT + DHT²**		**0.579 (0.350 – 0.938)**<br>**1.230 (0.976 – 1.450)** [b]	**0.029**<br>**0.025**	**0.569 (0.342 – 0.930)**<br>**1.330 (0.996 – 1.436)** [b]	**0.026**<br>**0.037**	**0.551 (0.334 – 0.894)**<br>**1.689 (1.156 – 2.491)** [b]	**0.017**<br>**0.007**
**fDHT**		**0.613 (0.369 – 0.971)**	**0.046**	**0.618 (0.372 – 0.979)**	**0.049**	**0.613 (0.369 – 0.971)**	**0.046**
**T/DHT**		1.559 (0.692 – 3.605)	0.272	1.556 (0.688 – 3.591)	0.274	1.559 (0.692 – 3.605)	0.272
**ln(SHBG)**		1.207 (0.804 – 1.836)	0.370	1.234 (0.820 – 1.884)	0.320	1.207 (0.804 – 1.836)	0.370

[a] Number of participants after exclusion of sex hormone or SHBG measurements 3 SDs above/below the mean: T (n = 384), fT (n = 386), DHT (n = 383), fDHT (n = 386), T/DHT (n = 387), and ln(SHBG) (n = 390).

[b] β-estimates presented for linear and quadratic terms of DHT due to significant quadratic term. Non-linearity was investigated by introducing a quadratic term to the models for all exposures. Here, only significant quadratic terms (P<0.05) are reported.

Incident CKD defined using follow-up eGFR <60 ml/min/1.73m^2 in participants without prevalent CKD. ORs are per 1 sex-specific SD and estimated using logistic regression. Adjusted for baseline age, baseline eGFR, waist circumference, systolic blood pressure, usage of antihypertensive medication, non-HDL cholesterol, prevalent cardiovascular disease, usage of lipid-lowering medication, smoking status, physical activity, alcohol consumption, baseline diabetes status, and ln(CRP).

Abbreviations: CI: Confidence interval, CKD: Chronic kidney disease, CRP: C-reactive protein, DHT: Dihydrotestosterone, eGFR: Estimated glomerular filtration rate, OR: Odds ratio, T: Testosterone.

References

[1] BIOCRATES Life Sciences. Absolute*IDQ*™ Stero17 Kit. Increased confidence in steroid hormone analysis. https://biocrates.com/wp-content/uploads/2020/02/Biocrates_Stero17.pdf; 2019. [accessed 6 October 2020]

[2] Committee for Medicinal Products for Human Use (CHMP). Guideline on bioanalytical method validation. EMEA/CHMP/EWP/192217/2009 Rev. 1 Corr. 2. https://www.ema.europa.eu/en/documents/scientific-guideline/guideline-bioanalytical-method-validation_en.pdf; 2011. [accessed 6 October 2020]

[3] Breier M, Wahl S, Prehn C, Ferrari U, Sacco V, Weise M, et al. Immediate reduction of serum citrulline but no change of steroid prodile after initiation of metformin in individuals with type 2 diabetes. J Steroid Biochem. 2017; 174;114-9. https://doi.org/10.1016/j.jsbmb.2017.08.004

[4] N. A. Mazer, "A novel spreadsheet method for calculating the free serum concentrations of testosterone, dihydrotestosterone, estradiol, estrone and cortisol: with illustrative examples from men and women populations," (in eng), *Steroids,* vol. 74, no. 6, pp. 512-9, Jun 2009, doi: 10.1016/j.steroids.2009.01.008.

[5] S. Gershagen, K. Henningsson, and P. Fernlund, "Subunits of human sex hormone binding globulin. Interindividual variation in size," (in eng), *J Biol Chem,* vol. 262, no. 17, pp. 8430-7, Jun 15 1987.
